# ﻿A comprehensive checklist of the deciduous photinia genus *Pourthiaea* (Maleae, Rosaceae), with emphasis on their validity and typification

**DOI:** 10.3897/phytokeys.202.85822

**Published:** 2022-07-19

**Authors:** Yi-Lei Lou, Ze-Tao Jin, Dai-Kun Ma, Bin-Bin Liu

**Affiliations:** 1 College of Landscape Architecture, Zhejiang Agriculture and Forestry University, Hangzhou 311300, China Zhejiang Agriculture and Forestry University Hangzhou China; 2 College of Landscape Architecture, Beijing Forestry University, Beijing 100083, China Beijing Forestry University Beijing China; 3 Centre of Pear Engineering Technology Research, State Key Laboratory of Crop Genetics and Germplasm Enhancement, Nanjing Agricultural University, Nanjing 210095, China Nanjing Agricultural University Nanjing China; 4 State Key Laboratory of Systematic and Evolutionary Botany, Institute of Botany, Chinese Academy of Sciences, Beijing 100093, China Institute of Botany, Chinese Academy of Sciences Beijing China

**Keywords:** Lectotype, nomenclature, *Photinia*, *Stranvaesia*, taxonomy, typification

## Abstract

Due to the complicated evolutionary history in *Pourthiaea*, ninety-seven taxa have been described since 1784, and ninety-one of them are validly published taxa, five are naked names, and one is an invalid name. After a comprehensive and critical evaluation, 213 names have been published, including new combinations, new status, and new names; this may be due to the controversial taxonomic position of *Pourthiaea* in the apple tribe, Maleae. We herewith provide a taxonomic checklist of *Pourthiaea* for further taxonomic and evolutionary studies. We also lectotypify two taxa: Photiniaamphidoxavar.stylosa and P.glabravar.fokienensis.

## ﻿Introduction

*Pourthiaea* Decne. (Maleae, Rosaceae) is a small genus of deciduous shrubs or trees distributed from East to Southeast Asia (Kuan and Yu 1974; Gu and Spongberg 2003). The taxonomic identity of *Pourthiaea* has been controversial for decades, either as a section of *Photinia* Lindl. (Kuan and Yu 1974; Robertson et al. 1991; Gu and Spongberg 2003; Campbell et al. 2007; Potter et al. 2007), or a synonym of *Aronia* Medik. (Kalkman 2004), or a separate genus *Pourthiaea* (Iketani and Ohashi 1991; Guo et al. 2011; Liu et al. 2019, 2022). The controversial taxonomic status of *Pourthiaea* makes the published names unstable regarding generic membership. According to our comprehensive review in the present study, nearly 213 names, including new combinations, new statuses, and new names, exist in the literature, representing 91 validly published taxa. For example, a deciduous shrub species endemic to South China was described as *Photiniabenthamiana* Hance in 1866. This taxon was then transferred either to *Pourthiaea* as *P.benthamiana* (Hance) Nakai in 1916 or to *Stranvaesia* as *S.benthamiana* (Hance) Merr. in 1917, as well as *Pyrus* as *P.benthamiana* (Hance) M.F.Fay & Christenh. in 2018.

Several recent phylogenetic studies have struggled to resolve the phylogenetic relationship between *Pourthiaea* and its closely related genera. Guo et al. (2011) firstly confirmed the monophyly of *Pourthiaea* using two plastid regions (*trnL-trnF* and *psbA-trnH*) and nuclear ribosomal ITS sequences. Unfortunately, they did not resolve the phylogenetic relationships between *Pourthiaea* and its relatives due to limited informative sites and taxon sampling. Based on transcriptomic data, Xiang et al. (2017) recovered a sister relationship between *Pourthiaea* and a combined clade, including *Chaenomeles* Lindl., *Cydonia* Mill., and *Pseudocydonia* (C.K.Schneid.) C.K.Schneid. Furthermore, results from the whole plastome and/or plastid regions (Liu et al. 2019, 2020a, 2020b, 2022) supported a close relationship between *Pourthiaea* and part of *Malus* Mill., i.e., the Eastern North American and Mediterranean Europe clade (clade II of figure 5 in Liu et al. 2022). However, the nuclear phylogeny inferred from 785 single-copy nuclear genes in our recent study (Liu et al. 2022) supported a close relationship between *Pourthiaea* and a combined clade, *Cydonia* and *Pseudocydonia*, not including *Chaenomeles*, contradicting Xiang et al. (2017)’s result inferred from transcriptomic data. The discordant phylogenetic position between nuclear and plastid topologies indicated a possible chloroplast capture event in the evolutionary history of *Pourthiaea* (Liu et al. 2022). This series of phylogenetic and phylogenomic studies confidently resolved the monophyly and phylogenetic position of *Pourthiaea*. In addition, the monophyly of *Pourthiaea* was also supported by some morphological characters, such as deciduous leaves, warty peduncles and pedicels, Kribs’III-I heterogeneous rays in the wood, and clusters of stone cells surrounded by parenchymatous cells in the flesh of pomes (Iketani and Ohashi 1991; Lu et al. 1991; Zhang 1992).

Liu et al. (2019) provided a robust phylogeny of the *Photinia* complex using whole plastomes and entire nuclear ribosomal DNA (nrDNA) sequences with a comprehensive taxon sampling. They proposed a redefined generic circumscription of *Pourthiaea*, transferring two species and a variety of *Stranvaesia* Lindl. to *Pourthiaea*, i.e., *S.amphidoxa* C.K.Schneid., S.amphidoxavar.amphileia (Hand.-Mazz.) T.T.Yu, and *S.tomentosa* T.T.Yu & T.C.Ku. Unfortunately, Guo et al. (2020) repeated these new taxonomic combinations, resulting in three illegitimate later homonyms. Recent systematic studies confidently resolved the generic circumscription of *Pourthiaea*, including all members of the previously recognized Photiniasect.Pourthiaea C.K.Schneid. and three newly transferred taxa from *Stranvaesia* (Guo et al. 2011, 2020; Liu et al. 2019, 2020a, 2020b, 2022). Furthermore, species delimitation within *Pourthiaea* has been controversial. Recent taxonomic treatments in *Pourthiaea* have been proposed solely from morphological evidence, either narrowly circumscribed (Kuan and Yu 1974; Gu and Spongberg 2003) or broadly circumscribed (Liu and Hong 2016a, 2016b, 2017). However, our recent phylogenomic evidence did not support these two proposals (unpublished data), indicating the need for an updated species delimitation in *Pourthiaea*. Hybridization, allopolyploidy, and apomixis have promoted the diversification of the apple tribe Maleae (Campbell et al. 1991; Robertson et al. 1991; Kalkman 2004; Liu et al. 2022). These complex evolutionary processes extensively diversified the genetic and morphological resources and greatly challenged the species delimitation in *Pourthiaea*. It is still premature to confidently delimit species in *Pourthiaea*.

Evaluating all the names published in *Pourthiaea* is the first step and will be helpful for further species delimitation. In this study, we made a comprehensive checklist of *Pourthiaea*. It should be noted that the checklist defined in this study is a summary of taxa (97) related to *Pourthiaea* rather than the species recognized currently in *Pourthiaea*, and these taxa have been described under *Crataegus* L., *Myrtus* L., *Photinia*, *Pourthiaea*, *Pyrus*, *Viburnum* L., and *Stranvaesia*. This checklist will provide a solid foundation for future taxonomic and evolutionary studies. In the genomic age, integrating hundreds of nuclear genes, plastomes and morphological characters make it possible to explore species delimitation in lineages with complicated evolutionary histories (Liu et al. 2021, 2022; Su et al. 2021).

## ﻿Materials and methods

To comprehensively explore all the names published under *Pourthiaea* and other related genera, we took over nine years (2013–2021) for this taxonomic study. We reviewed all names in the online databases, such as Tropicos (https://www.tropicos.org), IPNI (https://www.ipni.org/), The Plant List (http://www.theplantlist.org/), as well as all the literature related to *Pourthiaea*. In addition, we evaluated the validity of all names based on the Shenzhen Code (Turland et al. 2018). A total of 568 type specimens were checked; they are from the following 54 herbaria: A, AU, BNU, BM, BO, CDBI, CSFI, E, GH, GXMI, GZAC, HBG, HENU, HGAS, HHBG, HIB, HIMC, HITBC, HTC, HUST, HX, HZU, IBK, IBSC, JJF, JN, JXAU, K, KUN, KYO, L, LBG, M, N, NAS, NKU, NTUF, NY, P, PE, PH, QTPMB, SM, SNU, SYS, SZ, TAI, TI, U, UPS, US, WUK, ZJFC, and ZM, and the herbarium code followed the Index Herbariorum (http://sweetgum.nybg.org/science/ih/) hosted by New York Botanical Garden.

## ﻿Results

We reviewed 213 names published in *Pourthiaea* and its related genera, and they belong to 91 validly published taxa, five naked names, and one invalid name. We also lectotypified two taxa, i.e., Photiniaamphidoxavar.stylosa and P.glabravar.fokienensis. All these names in *Pourthiaea* have been arranged chronologically in the following text.

### ﻿Taxonomic checklist

***Pourthiaea* Decne., Nouv. Arch. Mus. Par. Ser. I, x. 146. 1874.**

Lectotype, designated by Iketani and Ohashi (1991: 353): *Pourthiaeavillosa* (Thunb.) Decne. ≡ *Crataegusvillosa* Thunb.

**1. *Crataeguslaevis* Thunb., Fl. Jap. (Thunberg) 204. 1784.**

≡ *Photinialaevis* (Thunb.) DC., Prodr. [A. P. de Candolle] 2: 631. 1825.

≡ Photiniavillosa(Thunb.)DC.var.laevis (Thunb.) Dipp. ex C.K.Schneid., Ill. Handb. Laubholzk. i. 710. 1906.

≡ Pourthiaeavillosa(Thunb.)Decne.var.laevis (Thunb.) Stapf, Bot. Mag. tab. 9275. 1929.

≡ *Pourthiaealaevis* (Thunb.) Koidz., Fl. Symb. Orient.-Asiat. 52. 1930.

**Type**: Japan. *s. loc.*, *C.P. Thunberg s. n.* (holotype: UPS [accession no. 11864]!).

**2. *Crataegusvillosa* Thunb., Fl. Jap. (Thunberg) 204. 1784.**

≡ *Photiniavillosa* (Thunb.) DC., Prodr. [A. P. de Candolle] 2: 631. 1825.

≡ *Pourthiaeavillosa* (Thunb.) Decne., Nouv. Arch. Mus. Par. Ser. I, x. 147. 1874.

≡ Photinialaevis(Thunb.)DC.var.villosa (Thunb.) Koidz., Bot. Mag. (Tokyo) 39: 313. 1925.

≡ Pourthiaealaevis(Thunb.)Koidz.var.villosa (Thunb.) Koidz., Fl. Symb. Orient.-Asiat. 52. 1930.

**Type**: Japan. *s. loc.*, *C.P. Thunberg s. n.* (holotype: UPS [accession no. 11881]!).

**3. *Myrtuslaevis* Thunb., Fl. Jap. (Thunberg) 198. 1784.**

**Type**: Japan. *s. loc.*, *C.P. Thunberg s. n.* (holotype: UPS [accession no. 11735]!).

**4. *Photiniaarguta* Lindl., Edwards’s Bot. Reg. 23: sub t. 1956. 1837.**

≡ *Pourthiaeaarguta* (Lindl.) Decne., Nouv. Arch. Mus. Par. Ser. I, x. 147. 1874.

≡ Pourthiaeaarguta(Lindl.)Decne.var.wallichii Hook.f., Fl. Brit. India [J. D. Hooker] 2(5): 382. 1878. nom. illeg.

≡ *Sorbusarguta* (Lindl.) Zabel, Handb. Laubh. Benenn. 200. 1903, non T.T.Yu, Acta Phytotax. Sin. viii. 223. 1963.

≡ *Pyrusarguta* (Lindl.) M.F.Fay & Christenh., Global Fl. 4: 95. 2018.

**Type**: India. West Bengal: Pandua, *N. Wallich 672* (lectotype, designated by Liu and Hong (2017: 18): K [barcode K000758328]!; isolectotypes: BM [barcode BM000602136, BM000946990]!, E [barcode E00011328]!, K [barcode K000758327]!, M [barcode M0213888, M0213888]!). (Image of lectotype available from https://plants.jstor.org/stable/10.5555/al.ap.specimen.k000758328).

**5. *Photiniapustulata* Lindl., Edwards’s Bot. Reg. 23: sub t. 1956. 1837.**

≡ Pourthiaeaarguta(Lindl.)Decne.subsp.pustulata (Lindl.) B.B.Liu & D.Y.Hong, Phytotaxa 325(1): 21. 2017.

**Type**: China. “China prope Cantonem, Parkes” [Guangdong]. *Parkes s. n.* (holotype: K [barcode K000758265]!). (Image of holotype available from https://plants.jstor.org/stable/10.5555/al.ap.specimen.k000758265).

**6. *Stranvaesiadigyna* Siebold & Zucc., Abh. Math.-Phys. Cl. Königl. Bayer. Akad. Wiss. 4(2): 129. 1845.**

**Type**: Japan. *H. Bürger s. n.* (lectotype, designated by Akiyama et al. (2014: 296): M [barcode M-0154027]!; isolectotypes: M [barcode M-0154026]!, M [barcode M-0154028]!). (Image of lectotype available from https://plants.jstor.org/stable/10.5555/al.ap.specimen.m0154027).

**7. *Photiniabenthamiana* Hance, Ann. Sci. Nat., Bot. sér. 5, 5: 213. 1866.**

≡ *Pourthiaeabenthamiana* (Hance) Nakai, Bot. Mag. (Tokyo) 30: 24. 1916.

≡ *Stranvaesiabenthamiana* (Hance) Merr., Philipp. J. Sci., C 12: 105. 1917.

≡ *Pyrusbenthamiana* (Hance) M.F.Fay & Christenh., Global Fl. 4: 98. 2018.

**Type**: China. Guangdong: Whampoa [Huangpu], April 1865, *H.F. Hance 1501* (lectotype (selected by Vidal (1968), first step; second step, designated by Liu and Hong (2017: 21)): K [barcode K000758275]!; isolectotypes: K [barcode K000758280]!, P [barcode P02143118, P02143182]!, U [barcode U0005861]!). Whampoa [Huangpu], May 1860, *H.F. Hance 1501* (syntype: K [barcode K000758276]!). ibidem, 20 September 1869, *H F. Hance 1501* (syntypes: GH [barcode 00045596]!, K [barcode K000758277]!). (Image of lectotype available from https://plants.jstor.org/stable/10.5555/al.ap.specimen.k000758275).

**8. Photiniavillosa(Thunb.)DC.var.formosana Hance, Ann. Sci. Nat., Bot., sér. 5 5: 212. 1866.**

≡ *Pourthiaeaformosana* (Hance) Koidz., Acta Phytotax. Geobot. 3(3): 147. 1934.

**Type**: China. Taiwan: Formosa [Taiwan]: Tamsuy [Danshui District], April 1864, *R. Oldham 146* (holotype: BM [barcode BM000602134]!; isotype: P [barcode P02143147]!). (Image of holotype available from https://plants.jstor.org/stable/10.5555/al.ap.specimen.bm000602134).

**9. *Pourthiaeacalleryana* Decne., Nouv. Arch. Mus. Par. Ser. I, x. 147. 1874.**

≡ *Photiniacalleryana* (Decne.) Cardot, Bull. Mus. Natl. Hist. Nat. xxvi. 568. 1920.

≡ *Pyrusdecaisnei* M.F.Fay & Christenh., Global Fl. 4: 99. 2018.

**Type**: China. Macao [Aomen], 1844, *J. Callery 97* (lectotype, designated by Liu and Hong (2017: 22): P [barcode P02143186]!; isolectotype: P [barcode P02143187]!). (Image of lectotype available from https://plants.jstor.org/stable/10.5555/al.ap.specimen.p02143186).

**10. *Pourthiaeacoreana* Decne., Nouv. Arch. Mus. Par. Ser. I, x. 148. 1874.**

≡ Pourthiaeavillosa(Thunb.)Decne.var.coreana (Decne.) Nakai, Bot. Mag. (Tokyo) 30: 25. 1916.

≡ Photiniavillosa(Thunb.)DC.var.coreana (Decne.) Rehder, J. Arnold Arbor. 2: 45. 1920.

**Type**: Japan. “Tsu-sima Island, St. of COREA”, 1859, *C. Wilford s. n.* (lectotype, designated by Liu and Hong (2016a: 211): P [barcode P03650663, excluding Corea. Port Chusan, 1859, *C. Wilford s. n.*]!; isolectotypes: GH [barcode 00026817, excluding Corea. Port Chusan, 1859, *C. Wilford s. n.*]!, P [barcode P03650621, excluding Japonia. Yokohama, 1862, *s. coll. s. n.*]!). (Image of lectotype available from https://science.mnhn.fr/institution/mnhn/collection/p/item/p03650663).

**11. *Pourthiaeacotoneaster* Decne., Nouv. Arch. Mus. Par. Ser. I, x. 149. 1874.**

≡ *Photiniacotoneaster* (Decne.) Cardot, Bull. Mus. Natl. Hist. Nat., xxvi. 569. 1920.

**Type**: Japan. *s. loc.*, *H. Zollinger 549* (lectotype, designated by Liu and Hong (2016a: 209): P [barcode P02143213]!; isolectotype: P [barcode P03650725]!). (Image of lectotype available from https://plants.jstor.org/stable/10.5555/al.ap.specimen.p02143213).

**12. *Pourthiaeahookeri* Decne., Nouv. Arch. Mus. Par. Ser. I, x. 148. 1874.**

≡ Pourthiaeaarguta(Lindl.)Decne.var.hookeri (Decne.) Hook.f., Fl. Brit. India [J. D. Hooker] 2(5): 382. 1878.

≡ *Photiniahookeri* (Decne.) Merr., Brittonia 4: 82. 1941.

≡ PhotiniaargutaLindl.var.hookeri (Decne.) J.E.Vidal, Adansonia, n.s. 5: 229. 1965.

**Type**: India. Sikkim, 1855, *J.D. Hooker & T. Thomson 652* (holotype: P [barcode P02143252]!). (Image of holotype available from https://plants.jstor.org/stable/10.5555/al.ap.specimen.p02143252).

**13. *Pourthiaealucida* Decne., Nouv. Arch. Mus. Par. Ser. I, x. 148. 1874.**

≡ *Photinialucida* (Decne.) C.K.Schneid., Ill. Handb. Laubholzk. i. 710. 1906.

≡ *Pyruspourthiaea* M.F.Fay & Christenh., Global Fl. 4: 116. 2018.

**Type**: China. Formosa [Taiwan]: Tamsuy [Danshui District], 1864, *R. Oldham 99* (lectotype, designated by Liu and Hong (2017: 20): P [barcode P02143146]!; isolectotypes: P [barcode P02143145, excluding the fruit branches]!, K [barcode K000758283]!, K [barcode K000758284]!). ibidem, *R. Oldham 99* (syntypes: P [barcode P02143145, excluding the flowering branch]!, K [barcode K000758284]!). (Image of lectotype available from https://plants.jstor.org/stable/10.5555/al.ap.specimen.p02143146).

**14. *Pourthiaeaoldhamii* Decne., Nouv. Arch. Mus. Par. Ser. I, x. 149. 1874.**

**Type**: Japan. Yokohama, 1863, *R. Oldham 242* (holotype: P [barcode P02143122]!, isotypes: GH [barcode 00026818]!, M [barcode M-0213882]!). 1853–56, *C. Wright s. n.* (syntype: P [barcode P02143123]!). (Image of holotype available from https://plants.jstor.org/stable/10.5555/al.ap.specimen.p02143122).

**15. *Pourthiaeasalicifolia* Decne., Nouv. Arch. Mus. Par. Ser. I, x. 148. 1874.**

≡ Pourthiaeaarguta(Lindl.)Decne.var.salicifolia (Decne.) Hook.f., Fl. Brit. India [J. D. Hooker] 2(5): 382. 1878.

≡ *Photiniasalicifolia* (Decne.) C.K.Schneid., Ill. Handb. Laubholzk. i. 709. 1906. “later homonym”, non C.Presl, Abh. Königl. Böhm. Ges. Wiss. ser. 5, 6: 564. 1851.

≡ PhotiniaargutaLindl.var.salicifolia (Decne.) J.E.Vidal, Adansonia, n.s. 5: 229. 1965.

≡ Pourthiaeaarguta(Lindl.)Decne.var.salicifolia (Decne.) Iketani & H.Ohashi, J. Jap. Bot. 66(6): 353. 1991. “later homonym”, non (Decne.) Hook.f., Fl. Brit. India [J. D. Hooker] 2(5): 382. 1878.

**Type**: Bangladesh. “East Bengal” or India. [Khasia] Meghalaya: Khasi, *W. Griffith 2099* (lectotype, designated by Vidal (1968: 51): P [barcode P02143251]!; isolectotype: K [barcode K000758337]!). (Image of lectotype available from https://plants.jstor.org/stable/10.5555/al.ap.specimen.p02143251).

**16. *Pourthiaeathunbergii* Decne., Nouv. Arch. Mus. Par. Ser. I, x. 149. 1874.**

**Type**: Japan. Nagasaki, 1862, *R. Oldham s. n.* (holotype: P [barcode P02143124]!). (Image of holotype available from https://plants.jstor.org/stable/10.5555/al.ap.specimen.p02143124).

**17. *Pourthiaeazollingeri* Decne., Nouv. Arch. Mus. Par. Ser. I, x. 149. 1874.**

≡ Photiniavillosa(Thunb.)DC.var.zollingeri (Decne.) C.K.Schneid., Ill. Handb. Laubholzk. i. 710. 1906.

≡ Pourthiaeavillosa(Thunb.)Decne.var.zollingeri (Decne.) Nakai, Bot. Mag. (Tokyo) 30: 25. 1916.

≡ Pourthiaealaevis(Thunb.)Koidz.var.zollingeri (Decne.) Koidz., Fl. Symb. Orient.-Asiat. 52. 1930.

**Type**: Japan. *s. loc.*, *H. Zollinger 548* (lectotype, designated by Liu and Hong (2016a: 215): P [barcode P02143126]!; isolectotype: P [barcode P03240057]!). (Image of lectotype available from https://plants.jstor.org/stable/10.5555/al.ap.specimen.p02143126).

**18. *Stranvaesiacalleryana* Decne., Nouv. Arch. Mus. Par. Ser. I, x. 179. 1874.**

**Type**: China. Macao [Aomen], 1844, *J. Callery 38* (lectotype, designated by Liu and Hong (2017: 22): P [barcode P02143183]!; isolectotypes: P [barcode P02143184]!, P [barcode P02143185]!). (Image of lectotype available from https://plants.jstor.org/stable/10.5555/al.ap.specimen.p02143183).

**19. *Photiniamollis* Hook.f., Fl. Brit. India [J. D. Hooker] 2(5): 381. 1878.**

**Type**: India. Sikkim Himalaya, at lower elevation, *J.D. Hooker s. n.* (holotype: K [barcode K000758335]!). Darjiling [Darjeeling], April 1876, *J.S. Gamble 451* (syntype: K [barcode K000758332]!). ibidem, *J.S. Gamble 451A* (syntype: K [barcode K000758333]!). ibidem, *J.S. Gamble 451E* (syntype: K [barcode K000758334]!). (Image of holotype available from https://plants.jstor.org/stable/10.5555/al.ap.specimen.k000758335).

**20. Pourthiaeaarguta(Lindl.)Decne.var.latifolia Hook.f., Fl. Brit. India [J. D. Hooker] 2(5): 382. 1878.**

≡ *Photiniabirmanensis* C.K.Schneid., Ill. Handb. Laubholzk. i. 709. 1906. replacement name.

**Type**: Myanmar. Kachin, Myitkyina, Tanaing, Hookhoom valley [Hukawng valley], 27 March 1827, *W. Griffith 2103* (holotype: K [barcode K000758346]!; isotypes: GH [barcode 00045582]!, P [barcode P03665952]!). (Image of holotype available from https://plants.jstor.org/stable/10.5555/al.ap.specimen.k000758346).

**21. Pourthiaeaarguta(Lindl.)Decne.var.membranacea Hook.f., Fl. Brit. India [J. D. Hooker] 2(5): 382. 1878.**

**Type**: not designated.

**22. Pourthiaeaarguta(Lindl.)Decne.var.parvifolia Hook.f., Fl. Brit. India [J. D. Hooker] 2(5): 382. 1878.**

**Type**: not designated.

**23. Photiniaglabra(Thunb.)Maxim.var.fokienensis Finet & Franch., Bull. Soc. Bot. France 47: 207. 1899.**

≡ *Photiniafokienensis* (Finet & Franch.) Franch., Bull. Mus. Natl. Hist. Nat. xxvi. 570. 1920.

≡ *Pourthiaeafokienensis* (Finet & Franch.) Iketani & H.Ohashi, J. Jap. Bot. 66(6): 353. 1991.

≡ *Pyrusfokienensis* (Finet & Franch.) M.F.Fay & Christenh., Global Fl. 4: 104. 2018.

**Type**: China. Fujian: Wuyishan County, Mount Wuyi, Kuatun [Guadun village], May 1998, *J.D.D. La Touche s.n.* (lectotype, designated here: P [barcode P02143166]!; isolectotypes: A [barcode 00026744, 00026745, 00026746]!, K [barcode K000758282, K000758354]!, L [barcode L0019504]!, MA [barcode MA628501, MA631213]!, P [barcode P02143167, P02143168]!). (Image of lectotype available from https://plants.jstor.org/stable/10.5555/al.ap.specimen.p02143166).

**24. *Pourthiaeaparvifolia* E.Pritz. ex Diels, Bot. Jahrb. Syst. 29(3–4): 389. 1900.**

≡ *Photiniaparvifolia* (E.Pritz. ex Diels) C.K.Schneid., Ill. Handb. Laubholzk. i. 711. 1906.

≡ Pourthiaealaevis(Thunb.)Koidz.var.parvifolia (E.Pritz. ex Diels) Migo, Bull. Shanghai Sci. Inst. 14: 311. 1944.

≡ Photiniavillosa(Thunb.)DC.var.parvifolia (E.Pritz. ex Diels) P.S.Hsu & L.Chu Li, Acta Phytotax. Sin. 18(3): 264. 1980.

≡ Pourthiaeavillosa(Thunb.)Decne.var.parvifolia (E.Pritz. ex Diels) Iketani & H.Ohashi, J. Jap. Bot. 66(6): 354. 1991.

≡ *Pyrusfantabulosa* M.F.Fay & Christenh., Global Fl. 4: 103. 2018.

**Type**: China. Prov. de Hupeh. [Hubei]: Ichang, 1885–88, *A. Henry 5830* (lectotype, designated by Liu and Hong (2016a: 215): P [barcode P02143150]!; isolectotypes: A [barcode 00045590]!, E [barcode E00011320, E00285981]!, GH [barcode 00045587]!, P [barcode P02143151]!). Szech’uan [Sichuan]: nanch’uan [Nanchuan], Tao kuo kou, *C. Bock & A. von Rosthorn 211* (syntype: A [barcode 00045589]!); ibidem, 1885–88, *A. Henry 3002* (syntypes: P [barcode P03342556]!; image A [barcode 00045569, with fragments from B]!). (Image of lectotype available from https://plants.jstor.org/stable/10.5555/al.ap.specimen.p02143150).

**25. *Photiniabeauverdiana* C.K.Schneid., Bull. Herb. Boissier Ser. II. vi. 319. 1906. (31 March 1906).**

≡ *Pourthiaeabeauverdiana* (C.K.Schneid.) Hatus., Bull. Exp. For. Kyushu Univ. 3: 99. 1933.

≡ *Pourthiaeabeauverdiana* (C.K.Schneid.) Migo, Bull. Shanghai Sci. Inst. 14: 310. 1944, “later homonym”. nom. illeg. superfl.

≡ *Pyrusbeauverdiana* (C.K.Schneid.) M.F.Fay & Christenh., Global Fl. 4: 98. 2018.

**Type**: China. Szetschwan [Sichuan]: S. Wushan County “now belongs to Chongqing”, *A. Henry 5599* (lectotype, designated by Vidal (1968: 43): K [barcode K000758272]!; isolectotypes: E [barcode E00010993]!, P [barcode P03373765]!). (Image of lectotype available from https://plants.jstor.org/stable/10.5555/al.ap.specimen.k000758272).

**26. *Photiniabergerae* C.K.Schneid., Ill. Handb. Laubholzk. i. 709. 1906. (1 May 1906).**

≡ *Pourthiaeabergerae* (C.K.Schneid.) Iketani & H.Ohashi, J. Jap. Bot. 66(6): 353. 1991.

≡ *Pyrusbergerae* (C.K.Schneid.) M.F.Fay & Christenh., Global Fl. 4: 98. 2018.

**Type**: China. W. Hupei [Hubei]: Patung [Badong County], *E.H. Wilson 86* (lectotype, designated by Liu and Hong (2017: 19): K [barcode K000758281]!; isolectotypes: A [barcode 00026737]!, NY [barcode 00436111]!). (Image of lectotype available from https://plants.jstor.org/stable/10.5555/al.ap.specimen.k000758281).

**27. *Photinianotabilis* C.K.Schneid., Ill. Handb. Laubholzk. i. 711. 1906. (1 May 1906).**

≡ PhotiniabeauverdianaC.K.Schneid.var.notabilis (C.K.Schneid.) Rehder & E.H.Wilson, Pl. Wilson. 1(2): 188. 1912.

≡ Pourthiaeabeauverdiana(C.K.Schneid.)Hatus.var.notabilis (C.K.Schneid.) Hatus., Bull. Exp. For. Kyushu Univ. 3: 99. 1933.

**Type**: China. W. Hupei [Hubei]: *E.H. Wilson 359* (lectotype, designated by Liu and Hong (2017: 19): K [barcode K000758271, excluding the fruit branches]!; isolectotypes: A [barcode 00038570, excluding the fruit branches]!, E [barcode E00010995, excluding the fruit branches]!, K [barcode K000758270, excluding the fruit branches]!, P [barcode P02143174, excluding the fruit branches]!, US [barcode 00097501, excluding the fruit branches]!). W. Hupei [Hubei], *E. H. Wilson 359* (syntypes: A [barcode 00038570, excl. the branch in flowering phase]!, E [barcode E00010995, excl. the branch in flowering phase]!, K [barcode K000758270, excl. the branch in flowering phase; K000758271, excl. the branch in flowering phase]!, P [barcode P02143174, excl. the branch in fruiting phase]!, US [barcode 00097501, excl. the branch in flowering phase]!). (Image of lectotype available from https://plants.jstor.org/stable/10.5555/al.ap.specimen.k000758271).

**28. *Stranvaesiaamphidoxa* C.K.Schneid., Bull. Herb. Boissier Ser. II. vi. 319. 1906.**

≡ *Photiniaamphidoxa* (C.K.Schneid.) Rehder & E.H.Wilson, Pl. Wilson. (Sargent) 1(2): 190. 1912.

≡ *Pourthiaeaamphidoxa* (C.K.Schneid.) Stapf, Bot. Mag. 155: sub t. 9275. 1929.

≡ *Pyrusamphidoxa* (C.K.Schneid.) M.F.Fay & Christenh., Global Fl. 4: 95. 2018.

**Type**: China. Szetschwan [Sichuan]: 1885–1888, *A. Henry 5565* (holotype: K [barcode K000758302]; isotypes: E [barcode E00010990]!, GH [barcode 00033523]!, US [barcode 00097546]!). (Image of holotype available from https://plants.jstor.org/stable/10.5555/al.ap.specimen.k000758302).

**29. *Photiniacavaleriei* H.Lév., Repert. Spec. Nov. Regni Veg. 4: 334. 1907.**

**Type**: China. Kouy-Tchéou [Guizhou]: Ly-Po [Libo County], 10 May 1899, *J. Cavalerie 2631* (lectotype, designated by Liu and Hong (2017: 19): E [barcode E00010994]!; isolectotypes: image A [barcode 00045601]! with fragments from E [barcode E00010994], P [barcode P02143180]!). (Image of lectotype available from https://plants.jstor.org/stable/10.5555/al.ap.specimen.e00010994).

**30. *Pyrusmokpoensis* H.Lév., Repert. Spec. Nov. Regni Veg. 7: 200. 1909. “ *Pirus* ”.**

**Type**: South Korea. “Circa Mokpo, Maio 1907”, *U. Faurie 1556* (holotype: E [barcode E00011325]!; isotype: A [barcode 00032499]!). (Image of holotype available from https://plants.jstor.org/stable/10.5555/al.ap.specimen.a00032499).

**31. *Viburnumkomarovii* H.Lév. & Vaniot, Repert. Spec. Nov. Regni Veg. 9: 78. 1910.**

≡ *Photiniakomarovii* (H.Lév. & Vaniot) L.T.Lu & C.L.Li, Acta Phytotax. Sin. 38(3): 278, 2000.

**Type**: China. Kouy-Tchéou [Guizhou]: Ma-Jo, 8 October 1908, *J. Cavalerie 1303* (lectotype, designated by Lu (2000: 278): A [barcode 00045609]!; isolectotypes: E [barcode E00011319]!, E [barcode E00011318, excluding the flower branches], P [barcode P03342568]!). (Image of lectotype available from https://plants.jstor.org/stable/10.5555/al.ap.specimen.a00045609).

**32. *Photiniataiwanensis* Hayata, J. Coll. Sci. Imp. Univ. Tokyo xxx. Art. 1, 104. 1911.**

**Type**: China. Taiwan: Taipei, Taihoku, 15 November 1896, *T. Makino s. n.* (holotype: TI [barcode T00596]!).

**33. *Photinialancifolia* Rehder & E.H.Wilson, Pl. Wilson. (Sargent) 1(2): 191. 1912.**

**Type**: China. Yunnan: Meng-lieh [Menglian County], 1,100 m, *A. Henry 12833* (holotype: A [barcode 00045581]!; isotypes: E [barcode E00010992]!, K [barcode K000758269]!, NY [barcode 00436113]!, US [barcode 00097498]!). Puer, Szemao [Simao], 1,300 m, *A. Henry 13412* (paratype: A [barcode 00137805]!). (Image of holotype available from https://plants.jstor.org/stable/10.5555/al.ap.specimen.a00045581).

**34. *Photiniaschneideriana* Rehder & E.H.Wilson, Pl. Wilson. (Sargent) 1(2): 188. 1912.**

≡ *Pourthiaeaschneideriana* (Rehder & E.H.Wilson) Iketani & H.Ohashi, J. Jap. Bot. 66(6): 354. 1991.

≡ *Pyrusschneideriana* (Rehder & E.H.Wilson) M.F.Fay & Christenh., Global Fl. 4: 120. 2018.

**Type**: China. Western Hupeh [Hubei]: Changyang Hsien, woodlands, 1300–1600 m, May 1907, *E.H. Wilson 476* (lectotype, designated by Liu and Hong (2017: 20): A [barcode 00045605]!; isolectotypes: A [barcode 00045585, excluding the fruit branches and the material in the packet]!, E [barcode E00011322, excluding the fruit branches and the material in the packet]!, GH [barcode 00038558]!, HBG [barcode HBG511071, excluding the fruit branches]!, K [barcode K000758288]!, K [barcode K000758289, excluding the fruit branches and the material in the packet]!, US [barcode 00097503, excluding the fruit branches]!). Changyang, woodlands, October 1907, *E.H. Wilson 476* (syntypes: A [barcode 00045585, excl. the branch in flowering phase]!, E [barcode E00011322, excl. the branch in flowering phase and the inflorescences from packet; E00284666]!, HBG [barcode HBG-511071, excl. the branch in flowering phase]!, K [barcode K000758289, excl. the branch in flowering phase and the inflorescences from packet]!, PE [barcode 01656469]!, SYS [barcode SYS00075135]!, US [barcode 00097503, excl. the branch in flowering phase]!). Chang-lo Hsien, woods, 1,000‒1,500 m, May 1907, *E.H. Wilson 2973* (paratypes: HBG [barcode HBG-511072]!, SYS [barcode SYS00075134]!). (Image of lectotype available from https://plants.jstor.org/stable/10.5555/al.ap.specimen.a00045605).

**35. *Photiniasubumbellata* Rehder & E.H.Wilson, Pl. Wilson. (Sargent) 1(2): 189. 1912.**

**Type**: China. Western Hupeh [Hubei]: Changyang Hsien, 1300 m, 7 May 1907, *E.H. Wilson 488* (holotype A [barcode 00038568, excluding the fruit branches]!; isotypes: E [barcode E00011321, excluding the fruit branches]!, HBG [barcode HBG511074, excluding the fruit branches]!, K [barcode K000758299, excluding the fruit branches]!, NY [barcode 00022711]!, US [barcode 00097505, excluding the fruit branches]!); ibidem, 7 October 1907, *E.H. Wilson 488* (paratypes: A [barcode 00038568, excl. the material collected in 7 May 1907, i.e. the branches in flowering phase]!, E [barcode E00011321, excl. the material collected in 7 May 1907, i.e. the branches in flowering phase]!, HBG [barcode HBG-511074, excl. the material collected in 7 May 1907, i.e. the branches in flowering phase]!, K [barcode K000758299, excl. the material collected in 7 May 1907, i.e. the branches in flowering phase]!, US [barcode 00097505, excl. the material collected in 7 May 1907, i.e. the branches in flowering phase]!); Western Hupeh, side of stream, 1,600–2,000 m, October 1907, *E.H. Wilson 398* (paratype: HBG [barcode HBG-511076]!). *A. Henry 7664* (paratype: P [barcode P03342557]!). Prov. Szech’uan [Sichuan], *A. Henry 5518* (paratype: P [barcode P03342562]!). (Image of holotype available from https://plants.jstor.org/stable/10.5555/al.ap.specimen.a00038568).

**36. Photiniavillosa(Thunb.)DC.var.sinica Rehder & E.H.Wilson, Pl. Wilson. 1(2): 186. 1912.**

≡ Pourthiaeavillosa(Thunb.)Decne.var.sinica (Rehder & E.H.Wilson) Migo, Bull. Shanghai Sci. Inst. 14: 311. 1944.

**Type**: China. Western Hupeh [Hubei]: Fang Hsien, 1300–1600 m, 4 June 1907, *E.H. Wilson 610* (lectotype, designated by Liu and Hong (2016a: 215): A [barcode 00045573, excluding the seeds in the packet]!; isolectotypes: A [barcode 00045572, excluding the fruit branches and the seeds in the packet]!, HBG [barcode HBG511081, excluding the fruit branches]!, K [barcode K000758296]!, US [barcode 00097507, excluding the fruit branches]!); ibidem, November 1907, *E.H. Wilson 610* (paratypes: A [barcode 00045572, excl. the materials collected in 4 June 1907, i.e. the branches in flowering phase]!, A [barcode 00045573, only including the seeds in the packet]!, HBG [barcode HBG-511081, excl. the materials collected in 4 June 1907, i.e. the branches in flowering phase]!, K [barcode K000758295]!, US [barcode 00097507, excl. the materials collected in 4 June 1907, i.e. the branches in flowering phase]!). Western Hupeh, woods, 1,000–1,300 m, May 1907, *E.H. Wilson 2972* (paratype: HBG [barcode HBG-511084]!). Xingshan, Western Hupeh, 1,000–1,500 m, May and October 1907, *E.H. Wilson 333* (paratype: HBG [barcode HBG-511083]!). W. Hupeh, *E.H. Wilson 714* (paratype: HBG [barcode HBG-511082]!). (Image of lectotype available from https://plants.jstor.org/stable/10.5555/al.ap.specimen.a00045573).

**37. *Pyrusbrunnea* H.Lév., Repert. Spec. Nov. Regni Veg. 10: 377. 1912. “ *Pirus* ”.**

≡ Pourthiaeavillosa(Thunb.)Decne.var.brunnea (H.Lév.) Nakai, Bot. Mag. (Tokyo) 30: 25. 1916.

≡ *Pourthiaeabrunnea* (H.Lév.) Nakai, Jap. J. Bot. 18: 617. 1942.

**Type**: South Korea. “Quelpaert, in silvis Hallaisan, 800m, October 1909”, *L. Taquet 2819* (holotype: E [barcode E00011324]!; isotype: A [barcode 00045571]!). (Image of holotype available from https://plants.jstor.org/stable/10.5555/al.ap.specimen.e00011324).

**38. PyrussinensisLindl.var.maximowicziana H.Lév., Repert. Spec. Nov. Regni Veg. 10: 377. 1912. “ *Pirus* ”.**

**Type**: South Korea. “Quelpaert, in silvis Haitchenam, aug. 1909”, *L. Taquet 2821* (holotype: E [barcode E00011326]!). (Image of holotype available from https://plants.jstor.org/stable/10.5555/al.ap.specimen.e00011326).

**39. PyrusspectabilisAitonvar.albescens H.Lév., Repert. Spec. Nov. Regni Veg. 10: 377. 1912. “*Pirus*”.**

≡ Pourthiaealaevis(Thunb.)Koidz.var.albescens (H.Lév.) Nakai, Bull. Natl. Sci. Mus. Tokyo 31: 62. 1952.

**Type**: South Korea. “Quelpaert, in silvis Hallaisan, 800m, jun. 1909”, *L. Taquet 2815* (holotype: E!; isotypes: A [barcode 00045577]!, image A [barcode 00045578] with a fragment ex E!).

**40. *Pyrusfeddei* H.Lév., Repert. Spec. Nov. Regni Veg. 12: 189. 1913. “ *Pirus* ”.**

**Type**: China. Kouy-Tchéou [Guizhou]: Pin-Fa [Pingfa Village, Guiding County], 8 October 1905, *J. Cavalerie & Fortunat 2533* (holotype: E [barcode E00010991]!; isotype: P [barcode P02143242]!). (Image of holotype available from https://plants.jstor.org/stable/10.5555/al.ap.specimen.e00010991).

**41. Photiniaamphidoxa(C.K.Schneid.)Rehder & E.H.Wilsonvar.stylosa Cardot, Notul. Syst. (Paris) 3: 377. 1914.**

**Type**: China. Kouy-tcheou [Guizhou]: “Tsin-gai, Kao-po”, 8 August 1903, *J. Cavalerie & Fortunat 1248* (lectotype, designated here: P [barcode P02143239]!). “Pin-fa, pentes boisées et humides”, 25 May 1905, *J. Cavalerie & Fortunat 2361* (syntypes: P [barcode P02143240, P02143241]!). “Ma-Jo, bo, comestille”, 24 June 1907, *J. Cavalerie 3107* (syntypes: P [barcode P02143243, P02143244]!). “Long-ly”, May 1908, *J. Cavalerie 3291* (syntypes: P [barcode P02143245, P02143246]!); ibidem, 8 August 1908, *J. Cavalerie 3839* (syntype: P [barcode P02143247]!). “bois près de Thong-oua”, May 1913, *J. Esquirol 4204* (syntype: P [barcode P02143248]!). (Note A) (Image of lectotype available from https://plants.jstor.org/stable/10.5555/al.ap.specimen.p02143239).

**Note A**: In the protologue, the specimen, *J. Cavalerie & Fortunat 2533*, was designated as the syntype of Photiniaamphidoxavar.stylosa by Cardot (1914); however, Léveillé (1913) had designated it as the type of *Pyrusfeddei*. Therefore, we exclude this specimen from the syntype of Photiniaamphidoxavar.stylosa. Considering the better condition of the specimen *J. Cavalerie & Fortunat 1248*, we designated it as the lectotype herein.

**42. PhotiniaargutaLindl.var.sinensis Cardot, Notul. Syst. (Paris) 3: 377. 1914.**

≡ *Cotoneasteresquirolii* H.Lév., Fl. Kouy-Tcheou 345. 1914–15. nom. illeg. superfl. (Note B)

≡ *Photiniaesquirolii* (H.Lév.) Rehder, J. Arnold Arbor. 17: 334. 1936.

**Type**: China. Kouy-tcheou [Guizhou]: “bois de Bai-gnin (Xingren County, Baling)”, 25 May 1911, *J. Esquirol 2624* (holotype: P [barcode P02143214]!; isotypes: image A [barcode 00026238]! with fragments from E [barcode E00011308], E [barcode E00011308]!). (Image of holotype available from https://plants.jstor.org/stable/10.5555/al.ap.specimen.p02143214).

**Note B**: Léveillé (1915) designated the specimen (*J. Esquirol 2624*) as the type of *Cotoneasteresquirolii*; however, this specimen has been designated as the type of Photiniaargutavar.sinensis by Cardot (1914). According to Article 52.1 & 52.2 in the International Code of Nomenclature for algae, fungi, and plants (Shenzhen Code) (Turland et al. 2018), *Cotoneasteresquirolii* definitely included the type of an earlier name, Photiniaargutavar.sinensis, so the former name is illegitimate and nomenclaturally superfluous and is to be rejected.

**43. PhotiniabeauverdianaC.K.Schneid.var.brevifolia Cardot, Notul. Syst. (Paris) 3: 378. 1914.**

≡ Pourthiaeabeauverdiana(C.K.Schneid.)Hatus.var.brevifolia (Cardot) Iketani & H.Ohashi, J. Jap. Bot. 66(6): 353. 1991.

**Type**: China. Western Hupeh [Hubei]: C. China, Shing-shan [Xingshan County] or Nanto, May 1900, *E.H. Wilson 794* (lectotype, designated by Liu and Hong (2017: 19): P [barcode P02143175]!; isolectotype: A [barcode 00045593]!). “Su-tchuen oriental: district de Tchen-keou-tin” [Chongqing: Chengkou County], *R.P. Farges s. n.* (syntypes: K [barcode K000758274]!, NY [barcode 00436110]!, P [barcode P02143177, P02143178, P02143179]!). C. China, Wushan, June 1900, *E.H. Wilson 1056* (syntypes: A [barcode 00045588]!, P [barcode P02143176]!). (Image of lectotype available from https://plants.jstor.org/stable/10.5555/al.ap.specimen.p02143175).

**44. PhotiniabenthamianaHancevar.glabrescens Cardot, Notul. Syst. (Paris) 3: 375. 1914.**

≡ Pourthiaeabenthamiana(Hance)Nakaivar.glabrescens (Cardot) Iketani & H.Ohashi, J. Jap. Bot. 66(6): 353. 1991.

**Type**: Vietnam. “Annam: massif du Lang-bian, entre Klou et Danhim, 900–1200m”, 19 February 1914, *A.J.B. Chevalier 30950* (lectotype, designated by Liu and Hong (2017: 21): P [barcode P02143197]!; isolectotypes: P [barcode P02143198, P02143199]!). (Image of lectotype available from https://plants.jstor.org/stable/10.5555/al.ap.specimen.p02143197).

**45. PhotiniabenthamianaHancevar.glabrescensCardotf.angustifolia Cardot, Notul. Syst. (Paris) 3: 376. 1914.**

**Type**: Vietnam. “Annam: massif du Lang-bian, Dalat, alt. 1400m”, 12 February 1914, *A.J.B. Chevalier 30739* (lectotype, designated by Liu and Hong (2017: 22): P [barcode P02143200]!; isolectotypes: P [barcode P02143201]!, P [barcode P02143202]!). (Image of lectotype available from https://plants.jstor.org/stable/10.5555/al.ap.specimen.p02143200).

**46. PhotiniabenthamianaHancevar.glabrescensCardotf.latifolia Cardot, Notul. Syst. (Paris) 3: 376. 1914.**

**Type**: Vietnam. “Annam: Plateau du Lang Bian, prov. du Haut-Donaï, 2500m”, *Jacquet 626* (lectotype, designated by Liu and Hong (2017: 22): P [barcode P02143188]!; isolectotype: P [barcode P02143189]!). (Image of lectotype available from https://plants.jstor.org/stable/10.5555/al.ap.specimen.p02143188).

**47. PhotiniabenthamianaHancevar.salicifolia Cardot, Notul. Syst. (Paris) 3: 376. 1914.**

≡ Pourthiaeabenthamiana(Hance)Nakaivar.salicifolia (Cardot) Iketani & H.Ohashi, J. Jap. Bot. 66(6): 353. 1991.

**Type**: Vietnam. “Annam: province de Thuan-tien, haut bassin du Bo-giang”, *P.A. Eberhardt 1999* (lectotype, designated by Vidal (1968: 47): P [barcode P02143194]!). “Annam: province de Thuan-tien, haut bassin du Bo-giang”, *P.A. Eberhardt 2738* (syntype: P [barcode P02143195, P02143196]!). (Image of lectotype available from https://plants.jstor.org/stable/10.5555/al.ap.specimen.p02143194).

**48. *Photiniabrevipetiolata* Cardot, Notul. Syst. (Paris) 3: 379. 1914.**

**Type**: China. “Kouy-tcheou: route de Pin-fa à Kouy-yang, haut plateau” [Guizhou: on the road from Pingfa village, Yunwu, Guiding County to Guiyang], *J. Cavalerie & Fortunat 2607* (holotype: P [barcode P02143212]!). (Image of holotype available from https://plants.jstor.org/stable/10.5555/al.ap.specimen.p021432).

**49. Photiniacalleryana(Decne.)Cardotvar.laosensis Cardot, Notul. Syst. (Paris) 3: 377. 1914.**

**Type**: Laos. “Bassin d’Attopeu”, March 1877, *J.H.A.J. Harmand 1131* (lectotype, designated by Liu and Hong (2017: 22): P [barcode P02143190]!; isolectotypes: P [barcode P02143191, P02143192, P02143193]!). (Image of lectotype available from https://plants.jstor.org/stable/10.5555/al.ap.specimen.p02143190).

**50. *Photiniafauriei* Cardot, Notul. Syst. (Paris) 3: 376. 1914.**

**Type**: China. Formosa [Taiwan]: Kelung [Jilong], 1914, *U.J. Faurie 81* (lectotype, designated by Liu and Hong (2017: 19): P [barcode P02143116]!; isolectotype: BM [barcode BM000629115]!). (Image of lectotype available from https://plants.jstor.org/stable/10.5555/al.ap.specimen.p02143116).

**51. *Photiniaparviflora* Cardot, Notul. Syst. (Paris) 3: 378. 1914.**

≡ *Pourthiaeaparviflora* (Cardot) Iketani & H.Ohashi, J. Jap. Bot. 66(6): 353. 1991.

≡ PhotiniaschneiderianaRehder & E.H.Wilsonvar.parviflora (Cardot) L.T.Lu & C.L.Li, Acta Phytotax. Sin. 38(3): 277. 2000.

≡ *Pyruseyola* M.F.Fay & Christenh., Global Fl. 4: 103. 2018.

**Type**: China. Kouy-tcheou [Guizhou]: “Kin-tchen-hia” [Longli County, Chengxia], 3 May 1907, *J. Cavalerie 3080* (holotype: P [barcode P02143181]!). (Image of holotype available from https://plants.jstor.org/stable/10.5555/al.ap.specimen.p02143181).

**52. PhotiniasubumbellataRehder & E.H.Wilsonvar.villosa Cardot, Notul. Syst. (Paris) 3: 379. 1914.**

≡ *Photiniacardotii* F.P.Metcalf, J. Arnold Arbor. 20(4): 440. 1939. replacement name.

**Type**: China. Fu-tschan [probably Ku-shan], 1883, *Popow s. n.* (holotype: P [barcode P02143152]!; isotype A [barcode 00045583]!-fragment ex P [barcode P02143152]). (Image of holotype available from https://plants.jstor.org/stable/10.5555/al.ap.specimen.p02143152).

**53. Photiniavillosa(Thunb.)DC.var.emergens Cardot, Notul. Syst. (Paris) 3: 378. 1914.**

**Type**: Japan. “montagne de Hakodate, octobre 1887”, *U. Faurie 3224* (lectotype, designated by Liu and Hong (2016a: 211): P [barcode P03650697]!; isolectotype: P [barcode P02143125]!). “Province d’Akita, octobre 1885”, *U. Faurie 1431* (syntype: P [barcode P03650628]!). (Image of lectotype available from https://science.mnhn.fr/institution/mnhn/collection/p/item/p03650697).

**54. *Photiniaimpressivena* Hayata, Icon. Pl. Formosan. 5: 67. 1915.**

≡ *Stranvaesiaimpressivena* (Hayata) Masam., Ann. Rep. Taihoku Bot. Gard. ii. 127. 1932.

≡ *Pourthiaeaimpressivena* (Hayata) Iketani & H.Ohashi, J. Jap. Bot. 66(6): 353. 1991.

≡ *Pyrusimpressivena* (Hayata) M.F.Fay & Christenh., Global Fl. 4: 108. 2018.

**Type**: China. Fokien [Fujian]: Fuzhou, Mt. Kozan, November 1909, *Nagasawa s. n.* (holotype: TI!; isotypes: A [barcode 00038572, 00038573]!).

**55. Pourthiaeavillosa(Thunb.)Decne.var.longipes Nakai, Bot. Mag. (Tokyo) 30: 25. 1916.**

≡ *Pourthiaealongipes* (Nakai) Nakai, Bot. Mag. (Tokyo) 48: 781. 1934.

**Type**: South Korea. Jeollanam-do: “Hab. in silvis Wangtô et montis Chirisan”, 20 June 1913, *T. Nakai s. n.* (lectotype, designated by Liu and Hong (2016a: 215): TI [barcode T00010502]!). in silvis montis Hakuyôzan, *S. Tate s. n.* (syntype: TI). in silvis montis Wangto, *T. Nakai 11358* (syntype: TI). Shinkinri insulae Gai-Rarôtô, *T. Nakai 11354* (syntype: TI). in silvis montis Hakuyôzan, Reisui, *T. Nakai 11355* (syntype: TI). in silvis montis Hakuyôzan, *T. Nakai 11356* (syntype: TI). in silvis montium Chirisan, *T. Nakai 139* (syntype: TI). in silvis montis Wangto, *T. Nakai 11358* (syntype: TI). Shinkinri insulae Gai-Rarôtô, *T. Nakai 11354* (syntype: TI). in silvis montis Hakuyôzan, Reisui, *T. Nakai 11355* (syntype: TI); in silvis montis Hakuyôzan, *T. Nakai 11356* (syntype: TI). in silvis montium Chirisan, *T. Nakai 139* (syntype: TI).

**56. *Cotoneasterblinii* H.Lév., Cat. Pl. Yun-Nan 229. 1917.**

≡ *Photiniablinii* (H.Lév.) Rehder J. Arnold Arbor. 17(4): 335. 1936.

≡ *Pourthiaeablinii* (H.Lév.) Iketani & H.Ohashi, J. Jap. Bot. 66(6): 353. 1991.

≡ *Pyrusblinii* (H.Lév.) M.F.Fay & Christenh., Global Fl. 4: 98. 2018.

**Type**: China. Kouy-tcheou [Guizhou]: “Goui-reou, lit même du fleuve”, 600 m, 2 October 1912, *J. Esquirol 3700* (holotype E [barcode E00010997]!; isotype: image A [barcode 00026738]! with a fragment ex E [barcode E00010997]. (Image of holotype available from https://plants.jstor.org/stable/10.5555/al.ap.specimen.e00010997).

**57. *Photiniaobliqua* Stapf, Bot. Mag. 149: sub t. 9008. 1924.**

≡ *Pourthiaeaobliqua* (Stapf) Iketani & H.Ohashi, J. Jap. Bot. 66(6): 354. 1991.

≡ *Pyrusobliqua* (Stapf) M.F.Fay & Christenh., Global Fl. 4: 114. 2018.

**Type**: China. Fokien [Fujian]: Foochow [Fuzhou], *W.R. Carles 839* (holotype: K [barcode K000758285]!; isotypes: image A [barcode 00038559]! with fragments from K [barcode K000758285]). (Image of holotype available from https://plants.jstor.org/stable/10.5555/al.ap.specimen.k000758285).

**58. Photiniaamphidoxa(C.K.Schneid.)Rehder & E.H.Wilsonvar.amphileia Hand.-Mazz., Symb. Sin. 7(3): 481. 1933.**

≡ StranvaesiaamphidoxaC.K.Schneid.var.amphileia (Hand.-Mazz.) T.T.Yu, Fl. Reipubl. Popularis Sin. 36: 214. 1974.

≡ Pourthiaeaamphidoxa(C.K.Schneid.)Rehder & E.H.Wilsonvar.amphileia (Hand.‐Mazz.) B.B.Liu & J.Wen, J. Syst. Evol. 57(6): 687. 2019.

≡ Pourthiaeaamphidoxa(C.K.Schneid.)Rehder & E.H.Wilsonvar.amphileia (Hand.‐Mazz.) W.Guo, W.B.Liao & LongY.Wang, Phytotaxa 447(2): 111. 2020. nom. illeg. superfl.

**Type**: China. Hunan: Wugang, Yunshan, 6–8 August 1917, 1150–1300 m, *H.R.E. Handel-Mazzetti 11205* (holotype: WU [barcode WU0059456]!; isotypes: A [barcode 00045586]!, IBSC [barcode 0362055]!, NY [barcode 00436109]!). ibidem, 15 April 1918, 1,100–1,190 m, *H.R.E. Handel-Mazzetti 11205* (syntype: WU [barcode WU0059455]!). April 1919, 1,100–1,300 m, *T.H. Wang 11205* (syntypes: WU [barcode WU0059454]!, A [barcode 00045592]!). (Image of holotype available from https://plants.jstor.org/stable/10.5555/al.ap.specimen.wu0059456).

**59. *Photiniahirsuta* Hand.-Mazz., Symb. Sin. Pt. vii. 481. 1933.**

≡ *Pourthiaeahirsuta* (Hand.-Mazz.) Iketani & H.Ohashi, J. Jap. Bot. 66(6): 353. 1991.

≡ *Pyrushirsuta* (Hand.-Mazz.) M.F.Fay & Christenh., Global Fl. 4: 107. 2018.

**Type**: China. Hunan: Changsha, “Hartlaubwalde des Yolu-schan bei Tschangcha, str. St., Sandstein, 100–300m”, 10 December 1917, *H.R.E. Handel-Mazzetti 11416* (holotype: WU [barcode WU0059450]!; isotype: A [barcode 00045606]!). (Image of holotype available from https://plants.jstor.org/stable/10.5555/al.ap.specimen.wu0059450).

**60. *Pourthiaeakankoensis* Hatus., Bull. Exp. For. Kyushu 3: 99. 1933.**

≡ Photiniaparvifolia(E.Pritz. ex Diels)C.K.Schneid.var.kankoensis (Hatus.) T.T.Yu & K.C.Kuan, Fl. Reipubl. Popularis Sin. 36: 259. 1974.

**Type**: China. Taiwan: Mt. Rohon, 900 m, November 1932, *S. Hatusima 793* (syntype FU).

**61. PourthiaeazollingeriDecne.var.yesoensis Nakai, Bot. Mag. (Tokyo) 48: 782. 1934.**

≡ Pourthiaealaevis(Thunb.)Koidz.var.yesoensis (Nakai) Nakai, Jap. J. Bot. 18: 618. 1942.

**Type**: Japan. Prov. Hidaka: Mt. Apoi, August 1928, *T. Nakai s. n.* (lectotype, designated by Liu and Hong (2016a: 215): TI [barcode 00002995]!). Prov. Hidaka: Samani, 18 June, *K. Miyabe s.n.* (syntype: TI [barcode T00002996]!).

**62. *Photiniaeuphlebia* Merr. & Chun, Sunyatsenia 2: 239. 1935.**

**Type**: China. Hainan: Qiongzhong, Fanyi village, 24 October 1932, 3000 m, *N.K. Chun & C.L. Tso 44134* (holotype: IBSC [barcode 0318867]!; isotypes: A [barcode 00026741]!, F [barcode f0068223f]!, IBK [barcode IBK00062249]!, IBSC [barcode 0318866]!, KUN [barcode 0641709]!, NY [barcode 00436119]!, P [barcode P02143171]!, PE [barcode 00336450]!, SYS [barcode SYS00095743]!, SZ [barcode 00197141]!, US [barcode 00097495]!, WIS [barcode v0255003WIS]!). Guangdong: Tsing Uen Dist. 4 April 1925, *F.A. McClure 1606* (paratypes: IBSC [barcode 0318815]!, SYS [barcode SYS00095741, SYS00095742]!). Zengcheng, Nankunshan, 1 May 1932, *W.T. Tsang 20396* [paratypes: IBSC [barcode 0318853, 0318858]!, NAS [barcode NAS00353222]!, PE [barcode 00336441]!, SYS [barcode SYS00074995, SYS00095748]!); ibidem, 3 April 1932, *W.T. Tsang 20072* (paratypes: IBSC [bardoce 0318864]!, KUN [barcode 0641700]!, NAS [barcode NAS00071254]!, PE [barcode 00336430, 00336432]!, SYS [barcode SYS00074996, SYS00095749]!). Meizhou, Jiaying, Yinnashan, 4 August 1932, *W.T. Tsang 21320* (paratypes: IBSC [barcode 0318859]!, NAS [barcode NAS00353223]!, PE [barcode 00336431]!, SYS [barcode SYS00095744]!). Dabu, Tonggushan, 8 September 1932, *W.T. Tsang 21703* (paratypes: IBSC [barcode 0318857]!, N [barcode 126077196]!, NAS [barcode NAS00353224, NAS00353231]!, PE [barcode 00336447, 00336448]!, SYS [barcode SYS00095747]!); Damaoshan, 26 June 1932, *W.T. Tsang 21033* (paratypes: IBSC [barcode 0318862]!, NAS [barcode NAS00374064, NAS00353218]!, PE [barcode 00336443, 00336425]!, SYS [barcode SYS00095745]!); ibidem, 3 July 1932, *W.T. Tsang 21060* (paratypes: IBSC [barcode 0318860]!, NAS [barcode NAS00353220, NAS00353221, NAS00353230]!, PE [barcode 00336445, 00336449]!, SYS [barcode SYS00095746]!).

**63. *Photiniatsaii* Rehder, J. Arnold Arbor. 19: 274. 1938.**

≡ *Pourthiaeatsaii* (Rehder) Iketani & H.Ohashi, J. Jap. Bot. 66(6): 354. 1991.

≡ *Pyrustsaii* (Rehder) M.F.Fay & Christenh., Global Fl. 4: 124. 2018.

**Type**: China. Yunnan: Shang-pa-hsien [Fugong County, Shangpa], 1500 m, 28 September 1933, *H.T. Tsai 54959* (holotype: A [barcode 00045570]!; isotypes: IBSC [barcode 0004377]!, KUN [barcode 313254]!, NAS [barcode NAS00071263]!, PE [barcode 00020610, 00020612]!). Shangpa, 1,500 m, 2 September 1933, *H.T. Tsai 54688* (paratypes: IBSC [barcode 0319885]!, KUN [barcode 0640795, specimen accession no. 0683743]!, NAS [barcode NAS00071262]!, PE [barcode 00337715, 00337717]!]; ibidem, 2,000 m, 24 October 1934, *H.T. Tsai 58945* (paratypes: IBSC [barcode 0004376]!, KUN [barcode 0640794]!, NAS [barcode NAS00354327]!, PE [barcode 00337716, 00337718]!). (Image of holotype available from https://plants.jstor.org/stable/10.5555/al.ap.specimen.a00045570).

**64. Photiniaamphidoxa(C.K.Schneid.)Rehder & E.H.Wilsonvar.kwangsiensis F.P.Metcalf, J. Arnold Arbor. 20(4): 442. 1939.**

**Type**: China. Guangxi: Sanjiang County, Lingwang Shan [Yuanbao Shan], 19 September 1933, 2100 m, *A.N. Steward & H.C. Cheo 1011* (holotype: A [barcode 00026736]!; isotypes: N [barcode 126077161, 126077161_1]!). (Image of holotype available from https://plants.jstor.org/stable/10.5555/al.ap.specimen.a00026736).

**65. PhotiniabeauverdianaC.K.Schneid.var.lohfauensis F.P.Metcalf, J. Arnold Arbor. 20(4): 440. 1939.**

**Type**: China. Kwantung [Guangdong]: Loh-fau [Luofu Mountain], 14 August 1917, *C.O. Levine 1377* (holotype: A [barcode 00045595]!). ibidem, *C.O. Levine 1381* (paratypes: AU?, A?); ibidem, *C.O. Levine 1553* (paratype: AU?). Lechang, 29 May 1929, *C.L. Tso 20832* (paratypes: IBSC [barcode 0004360, 0319644, 0319655]!). Yunan: Lung T’au Shan [Longtang Shan], 18 June 1924, *Lingnan (To and Ts’ang) 12503* (pratypes: PE [barcode 00337413, 00337414]!); ibidem, 7 June 1924, *Lingnan (To and Ts’ang) 12432* (IBSC [barcode 0319637]!). Xiamen: *H.H. Chung 6399* (paratypes: AU [barcode 010130, 010132]!). (Image of holotype available from https://plants.jstor.org/stable/10.5555/al.ap.specimen.a00045595).

**66. PhotiniamollisHook.f.var.angustifolia C.E.C.Fisch., Bull. Misc. Inform. Kew 7: 335. 1939.**

**Type**: Myanmar. Myitkyina District: Tanpangkha, about 3 miles from Seniku, on rocks beside the stream, 300 m, 22 March 1938, *C.W.D. Kermode 16608* (holotype: K (barcode K000758336)!). (Image of holotype available from https://plants.jstor.org/stable/10.5555/al.ap.specimen.k000758336).

**67. Pourthiaealaevis(Thunb.)Koidz.var.crassiuscula Nakai, Jap. J. Bot. 18: 619. 1942.**

**Type**: Japan. Prov. Sinano: in monte Togakusi, 11 July 1884, *Z. Mutamura s. n.* (lectotype, designated by Liu and Hong (2016a: 215): TI [barcode 00031786]!). Prov. Uzen: Yonezawa, October 1914, *G. Koidzumi* (paratype: TI [barcode T00031788]!). Prov. Simotuke: Tyûzenzi, 29 September 1879, *K. Sawada* (paratype: TI). Prov. Yamasiro: Hattyôdaira oppidi Kuta, 29 September 1934, *Y. Momiyama 660* (paratype: TI). Prov. Uzen: Yonezawa, October 1914, *G. Koidzumi s. n.* (syntype: TI [barcode T00031788]!).

**68. Pourthiaeavillosa(Thunb.)Decne.var.yokohamensis Nakai, Jap. J. Bot. 18: 618. 1942.**

**Type**: Japan. Yokohama, Musasi, 23 June 1932, *K. Hisauchi s. n.* (lectotype, designated by Liu and Hong (2016a: 215): TI [barcode 00010504]!). Yokohama, Musasi, 15 May 1933, *K. Hisauchi s. n.* (paratype: TI [barcode T00010505]!).

**69. *Pyrusmoiorum* A.Chev., Rev. Bot. Appl. Agric. Trop. xxii. 375. 1942.**

≡ *Photiniamoiorum* (A.Chev.) J.E.Vidal, Fl. Cambodge, Laos & Vietnam Fasc. 6, 48. 1968.

≡ *Pourthiaeamoiorum* (A.Chev.) Iketani & H.Ohashi, J. Jap. Bot. 66(6): 354. 1991.

≡ *Photiniapirocarpa* J.E.Vidal, Notul. Syst. (Paris) 13(4): 300. 1948. nom. illeg. superfl. (referring to the Note 15 in Liu and Hong (2017)).

**Type**: Laos. “Nord de Pakson plateau des Boloven, Pro. de Bassac”, 1300 m, 31 October 1928, *E. Poilane 16225* (lectotype (selected by Vidal (1968), first step; second step, designated by Liu and Hong (2017)): P [barcode P02143149]!; isolectotypes: A [barcode 00026801], K [barcode K000758355, K000758356)!, L [barcode L0019508]!, P [barcode P02143148, P02143204]!). (Image of lectotype available from https://plants.jstor.org/stable/10.5555/al.ap.specimen.p02143149).

**70. PhotiniabenthamianaHancevar.obovata H.L.Li, J. Arnold Arbor. 25(2): 208. 1944.**

≡ Pourthiaeabenthamiana(Hance)Nakaivar.obovata (H.L.Li) Iketani & H.Ohashi, J. Jap. Bot. 66(6): 353. 1991.

**Type**: China. Hainan: Lingshui County, 300 ft., 20 October 1935, *F.C. How 73904* (holotype: A [barcode 00045597]!; isotypes: IBSC [barcode 0318212]!, P [barcode P03240040]!). (Image of holotype available from https://plants.jstor.org/stable/10.5555/al.ap.specimen.a00045597).

**71. *Photinialancilimbum* J.E.Vidal, Notul. Syst. (Paris) 13(4): 298. 1948.**

**Type**: Vietnam. “Annam: Nui Bach Ma station d’altitude de Huê” [Hué, Bach Ma National Park], 1200–1500 m, 13 December 1940, *E. Poilane 31128* (lectotype, designated by Liu and Hong (2017: 22): P [barcode P02143249]!; isolectotypes: K [barcode K000758351]!, P [barcode P02143253]!). Lac Duong, “Annam: Nui Bach Ma station d’altitude de Huê” [Hué, Bach Ma National Park], 13 December 1940, *E. Poilane 31137* (syntypes: P [barcode P03373767, P03373771]!). “massif du Lang Bian” [Đà Lạt Plateau Liang Bian], 1,000–1,200 m, 29 April 1919, *A.J.B. Chevalier 40347* (syntypes: P [barcode P03373769, P03373770]!). “Annam: Nui Bach Ma station d’altitude près de Hué Grand cascade - Altitude 1,000 m” [Hué, Bach Ma National Park], 15 April 1939, *E. Poilane 29737* (syntypes: P [barcode P03373734, P03156772, P03373735]!). (Image of lectotype available from https://plants.jstor.org/stable/10.5555/al.ap.specimen.p02143249).

**72. PhotinialancilimbumJ.E.Vidalvar.petaloconstricta J.E.Vidal, Notul. Syst. (Paris) 13(4): 299. 1948.**

**Type**: Laos: “M. Xuong, prov. Luang Prabang”, 400 m, 26 March 1932, *E. Poilane 20517* (lectotype, designated by Liu and Hong (2017: 22): P [barcode P02143215]!; isolectotypes: K [barcode K000758352]!, P [barcode P02143216]!). (Image of lectotype available from https://plants.jstor.org/stable/10.5555/al.ap.specimen.p02143215).

**73. PhotinialancilimbumJ.E.Vidalvar.racemosa J.E.Vidal, Notul. Syst. (Paris) 13(4): 299. 1948.**

**Type**: Vietnam. “Tonkin: cours de la Rivière Noire en aval de Lai Chau”, 21 January 1938, *E. Poilane 27159* (lectotype, designated by Liu and Hong (2017: 15): P [barcode P02143217]!; isolectotypes: K [barcode K000758353]!, P [barcode P02143218]!). (Image of lectotype available from https://plants.jstor.org/stable/10.5555/al.ap.specimen.p02143217).

**74. PhotinialancilimbumJ.E.Vidalvar.turbinata J.E.Vidal, Notul. Syst. (Paris) 13(4): 299. 1948.**

**Type**: Vietnam. “Plateau d’altitude de Bach Ma, près Hué” [Hué, Bach Ma National Park], 16 June 1944, *J.E. Vidal 33A* (holotype: P [barcode P02143219]!). (Image of holotype available from https://plants.jstor.org/stable/10.5555/al.ap.specimen.p02143219).

**75. PhotinialancilimbumJ.E.Vidalvar.urceolocarpa J.E.Vidal, Notul. Syst. (Paris) 13(4): 299. 1948.**

≡ PhotiniaimpressivenaHayatavar.urceolocarpa (J.E.Vidal) J.E.Vidal, Fl. Cambodge, Laos & Vietnam 6: 51, pl. 6, f. 4–5. 1968.

≡ Pourthiaeaimpressivena(Hayata)Iketani & H.Ohashivar.urceolocarpa (J.E.Vidal) Iketani & H.Ohashi, J. Jap. Bot. 66(6): 354. 1991.

**Type**: Vietnam. “bords du torrent de Muong Xen, Km. 19 Route de Lao Cai à Cha Pa”, 1 August 1944, *J.E. Vidal 32* (lectotype, designated by Vidal (1968: 52): P [barcode P02143250]!). LAO CAI. “bords du torrent de Muong Xen, Km. 19 Route de Lao Cai à Cha Pa”, August 1943, *A. Pélelot 8407* (syntype: P [barcode P03342482]!). ibidem, 1 August 1944, *J.E. Vidal 32A* (syntypes: P [barcode P02143172, P02143173]!). (Image of lectotype available from https://plants.jstor.org/stable/10.5555/al.ap.specimen.p02143250).

**76. Pourthiaeavillosa(Thunb.)Decne.var.oblongifolia Murata, Acta Phytotax. Geobot. 15: 177. 1954.**

≡ Pourthiaeavillosa(Thunb.)Decne.var.laevis(Thunb.) Stapff.oblongifolia (Murata) Satomi, J. Phytogeogr. Taxon. 28(1): 32. 1980.

**Type**: Japan. “Honshiu Prov. Yamato: Inter Misen et Shakadake in Montibus Oomine”, 1750 m, 16 July 1954, *G. Murata & T. Shimizu 122* (holotype: KYO [barcode 00081202]!; isotype: KYO [barcode 00081203]!).

**77. *Photiniacallosa* Chun ex T.T.Yu & K.C.Kuan, Acta Phytotax. Sin. 8: 229. 1963.**

≡ *Pourthiaeacallosa* (Chun ex T.T.Yu & K.C.Kuan) Iketani & H.Ohashi, J. Jap. Bot. 66(6): 353. 1991.

≡ *Pyruscallosa* (Chun ex T.T.Yu & K.C.Kuan) M.F.Fay & Christenh., Global Fl. 4: 99. 2018.

**Type**: China. Guangdong: Xinyi County, Dadong, Shuiyinshan, 20 April 1932, *Z. Huang 32130* (holotype: IBSC [barcode 0004361]!; isotypes: IBK [barcode IBK00062047, IBK00062048]!, KUN [barcode 607112, 607113]!, PE [barcode 00004608, 00004609]!, SZ [barcode 00196906]!). Xinyi, Daguowei, Huochangping, 4,500 ft, 28 April 1932, *Z. Huang 32255* (paratypes: IBK [barcode IBK00062046, IBK00062049]!, IBSC [barcode 0318299]!, KUN [barcode 0641136]!, PE [barcode 00299777]!, SZ [barcode 00196905]!]. Fenshui’ao, 26 April 1931, *X.P. Gao 51361* (paratypes: IBK [barcode IBK00061932]!, IBSC [barcode 0318293, 0318294, 0318295, 0318296]!]. Fangcheng, 13 April 1956, *Guangdong Hepu District Expedition of CAS 2371* (paratypes: IBSC [barcode 0318234]!, PE [barcode 00299778]!. Longsheng, Pingshui, Taoshan, 11 August 1954, *Longsheng Expedition 157* (paratypes: IBK [barcode IBK00190806]!, IBSC [barcode 0318300]!). Shangsi, Me-kon, Sch-feng Dar Shan, S.Nanning, ca. 850 m, 3 November 1928, *R.C. Ching 8373* (paratypes: IBSC [barcode 0004362]!, NAS [barcode NAS00071251, NAS00352714, NAS00352716]!, PE [barcode 00299789]!). “Shap Man Taai Shan, near Iu Shan village, S. E. of Shang-sze, Kwangtung Border” [Shangsi, Shiwan Dashan], 2 June 1933, *W.T. Tsang 22441* (paratypes: IBK [barcode IBK00062034]!, IBSC [barcode 0318297]!, P [barcode P03373754]!, SYS [barcode SYS00074925]!). Shiwan Dashan, Denglong village, 9 September 1934, *W.T. Tsang 24222* (paratypes: IBSC [barcode 0318298]!, SYS [barcode SYS00074926]!). Wuming, Shanglin, Damingshan, the seventh district, Lahen, 22 August 1951, *T.S. Tsai 5335* (paratypes: IBK [barcode IBK00190807]!, IBSC [barcode 0318302]!). Yongfu, “Pai-shou district city, near Shih-lung ts’un” [Yongfu], 20–23 August 1937, *Y.W. Taam 25* (paratypes: IBSC [barcode 0318301]!, PE [barcode 00299784]!, SYS [barcode SYS00074927]!).

**78. PhotiniahirsutaHand.-Mazz.var.lobulata T.T.Yu, Acta Phytotax. Sin. 8(3): 231. 1963.**

**Type**: China. Fujian: Liancheng, Luodi, 24 October 1932, *Y. Ling 4167* (holotype: PE [barcode 00004605]!; isotypes: AU [barcode 010148]!, PE [barcode 00004604]!).

**79. *Photiniapilosicalyx* T.T.Yu, Acta Phytotax. Sin. viii. 231. 1963.**

≡ *Pourthiaeapilosicalyx* (T.T.Yu) Iketani & H.Ohashi, J. Jap. Bot. 66(6): 354. 1991.

≡ *Pyruspilosicalyx* (T.T.Yu) M.F.Fay & Christenh., Global Fl. 4: 116. 2018.

**Type**: China. Guizhou: Anlong County, Longshan, Qiaoling, 1,200 m, 9 June 1960, *Z.S. Zhang & Y.T. Zhang 4035* (holotype: PE [barcode 00934236]!; isotypes: IBSC [barcode 0335502]!, KUN [barcode 607037]!).

**80. *Photiniapodocarpifolia* T.T.Yu, Acta Phytotax. Sin. viii. 230. 1963.**

≡ *Pourthiaeapodocarpifolia* (T.T.Yu) Iketani & H.Ohashi, J. Jap. Bot. 66(6): 354. 1991.

≡ *Pyruspodocarpifolia* (T.T.Yu) M.F.Fay & Christenh., Global Fl. 4: 116. 2018.

**Type**: China. Guizhou: Luodian, Bamao, Dating, Hongshui River, 200 m, 8 April 1959, *Qiannan Expedition 408* (lectotype, designated by Liu and Hong (2016b: 225): PE [barcode 00004600]!; isolectotypes: HGAS [barcode 021090]!, KUN [barcode 606957, 747408]!, PE [barcode 00020608, 01498393, 01498394]!). Hongshui River, riverside, 8 April 1959, *Qiannan Expedition 709* (paratypes: HGAS [barcode 021089]!, KUN [barcode 606956]!, NAS [barcode 00071260]!, PE [barcode 00337090, 00337091, 01651162]!). Guangxi: Baise City, Yangxu Zhen, 280 m, 25 December 1955, *Baise Expedition 1534* (paratypes: IBK [barcode IBK-00190813]!, IBSC [barcode 0004370]!, KUN [barcode 606955]!, NAS [barcode 00071259]!, PE [barcode 00337094]!). Petseu, 1914, *J. Cavalerie 4283* (paratype: P [barcode P03342555]!). ibidem, 1900–1920, *J. Cavalerie 4498* (paratypes: K [barcode K000758287]!, P [barcode P03373739]!). Longlin County, Gebu Xiang, Wuchong Village, on the rocks by river, 13 May 1957, *C.F. Liang 32325* (paratypes: IBK [barcode IBK00190814]!, IBSC [barcode 0004369]!). Tian’e County, Baiyang Zhen, Hongshui River, riversides, 150 m, 27 March 1960, *Guizhou Expedition 14* (paratypes: IBK [barcode 00062433]!, IBSC [barcode 0319277]!, NAS [barcode 00071258]!, PE [barcode 00337093]!). Liupai Zhen, 21 May 1957, *Z. Huang 43389* (paratypes: IBK [barcode IBK00190812]!, IBSC [barcode 0319273]!, KUN [barcode 606954]!). Tianlin County, on the way from Leli Xiang to Lizhou Xiang, on the rocks in valley, 22 November 1957, *Z.Q. Zhang 10921* (paratypes: IBK [barcode IBK00062427]!, IBSC [barcode 0319272]!).

**81. *Pourthiaeachingshuiensis* T.Shimizu, Journ. Fac. Text. Sci. & Techn., Shinshu Univ. No. 36, Biol., No. 12 (Stud.Limest. Fl. Jap. & Taiwan, Pt. 2) 36. 1963; et in Acta Phytotax. & Geobot.,Kyoto, xxi. 20. 1964.**

≡ *Photiniachingshuiensis* (T.Shimizu) T.S.Liu & H.J.Su, Fl. Taiwan 3: 74. 1977.

≡ Photiniaparvifolia(E.Pritz. ex Diels)C.K.Schneid.var.chingshuiensis (T.Shimizu) S.S.Ying, Coloured Illustr. Fl. Taiwan 1: 358. 1985.

≡ Pourthiaeavillosa(Thunb.)Decne.var.chingshuiensis (T.Shimizu) Iketani & H.Ohashi, J. Jap. Bot. 66(6): 354. 1991.

≡ *Pyruschingshuiensis* (T.Shimizu) M.F.Fay & Christenh., Global Fl. 4: 100. 2018.

**Type**: China. Taiwan: Hualien, Mt. Chingshui, 600–1400 m, 29 March 1961, *T. Shimizu & M.T. Kao 11749* (holotype: TAI [barcode 055602]!; isotypes: KYO [barcode KYO00022343]!, SHIN [barcode 155840]!, TI [barcode TI00031789]!).

**82. *Stranvaesiatomentosa* T.T.Yu & T.C.Ku, Acta Phytotax. Sin. 13(1): 102. 1975.**

≡ *Pyrusganymedes* M.F.Fay & Christenh., Global Fl. 4: 104. 2018.

≡ *Pourthiaeatomentosa* (T.T.Yu & T.C.Ku) B.B.Liu & J.Wen, J. Syst. Evol. 57(6): 687. 2019.

≡ *Pourthiaeatomentosa* (T.T.Yu & T.C.Ku) Long Y.Wang, W.Guo & W.B.Liao, Phytotaxa 447(2): 111. 2020. nom. illeg. superfl.

**Type**: China. Chongqing: Nanchuan, Jinfoshan, Dafengpo, 1,350 m, 9 July 1964, *K.C. Kuan et al. 1319* (holotype: PE [barcode 00026319]!; isotypes: CDBI [barcode CDBI0172158]!, PE [barcode 00026320]!). Nanchuan, Guandi, 650 m, 3 May 1957, *G.F. Li 60885* (paratypes: HIB [barcode 0037841]!, KUN [barcode 0643094]!, PE [barcode 00739363]!, SZ [barcode 00215295, 00215296]!). Beibei, Jinyunshan, NE of Qingyunzhai, 820 m, 31 May 1956, *Sichuan-Guizhou Expedition 471* (paratype: PE [barcode 00739360]!); Jinyunshan, Pokongta, 780 m, *T.H. Tu 5110* (paratypes: IBSC [barcode 0335389]!, PE [barcode 00739353]!); Jinyunshan, 670 m, 29 April 1930, *K.S. Hao 61* (paratype: PE [barcode 00739362]!); ibidem, 6 July 1930, *S.F. Zhang 659* (paratype: PE [barcode 00739352]!); ibidem, 11 January 1940, *C. Pei 7516* (paratype: PE [barcode 00739361]!).

**83. Photiniavillosa(Thunb.)DC.var.tenuipes P.S.Hsu & L.ChuLi, Acta Phytotax. Sin. 18(3): 264. 1980.**

≡ Photiniaparvifolia(E.Pritz. ex Diels)C.K.Schneid.var.tenuipes (P.S.Hsu & L.Chu Li) P.L.Chiu, J. Zhejiang Forest. Coll. 4(1): 67. 1987.

≡ Pourthiaeavillosa(Thunb.)Decne.var.tenuipes (P.S.Hsu & L.ChuLi) Iketani & H.Ohashi, J. Jap. Bot. 66(6): 354. 1991.

**Type**: China. Zhejiang: Lin’an, Changhua, Kuliwan, 20 May 1958, *X.Y. He 28542* (holotype: FUS, isotypes: IBSC [barcode 0335093]!, PE [barcode 00394001]!); Jingning: Yingchuan, Laoyue, in the bushes of the valley, 9 May 1959, *S.Y. Zhang 4928* (paratypes: FUS, HHBG [barcode HZ016336]!, KUN [barcode 608586]!, NAS [barcode NAS00353328, NAS00353262]!; PE [barcode 00337971]!); Jingning, Dajun, Quankeng, roadsides of the slope, 9 May 1959, *S.Y. Zhang 5131* (paratypes: FUS, HHBG [barcode HZ016335]!, NAS [barcode NAS00353274, NAS00353275, NAS00353278]!; PE [barcode 00337983]!; WUK [barcode WUK-0264321]!); Lishui, Dagangtou, Xiaojing, roadsides of the slope and in the woods beside the stream, 28 July 1959, *S.Y. Zhang 6041* (paratypes: FUS, HHBG [barcode HZ016334]!; KUN [barcode 608587]!, NAS [barcode NAS00353302, NAS00353270]!, PE [barcode 00337997]!); Lishui, Yunfeng, roadsides and in the woods on the slope, 17 August 1959, *S.Y. Zhang 6387* (paratypes: FUS, HHBG [barcode HZ016346]!, KUN [barcode 608588]!, NAS [barcode NAS00353325]!, PE [barcode 00337995]!); ibidem, 21 August 1959, *S.Y. Zhang 6700* (paratypes: FUS, HHBG [barcode HZ016345]!, KUN [barcode 608594], NAS [barcode NAS00353326, NAS00353327]!, PE [barcode 00337994]!); Lin’an, Changhua, south-east of Jinkeng in the woods beside the stream, 25 May 1958, *X.Y. He 28699* (paratypes: FUS, HHBG [barcode HZ016316]!, NAS [barcode NAS00353263]!, PE [barcode 00337972]!); Lin’an, Changhua, the centre part of Kuliwan in the woods on a north-western slope, 27 May 1958, *X.Y. He 28745* (paratypes: FUS, HHBG [barcode HZ016325]!, NAS [barcode NAS00353323, NAS00353264, NAS00353267]!, PE [barcode 00338000]!); Longquan, Jinxi, Xiaozhuang, in the woods in the valley or beside the stream, 20 September 1959, *S.Y. Zhang 6869* (paratypes: FUS, HHBG [barcode HZ016338]!, KUN [barcode 608589]!, NAS [barcode NAS00353330, NAS00353331]!, PE [barcode 00337982]!); Pingyang, Juxi, in the woods of the valley, 26 June 1959, *S.Y. Zhang 5805* (paratypes: FUS, KUN [barcode 608591]!, NAS [barcode NAS00353277, NAS00353298]!, PE [barcode 00337985]!); Rui’an, Shiyang, Baiyanwan, in the woods beside the stream, 27 July 1959, *S.Y. Zhang 5479* (paratypes: FUS, HHBG [barcode HZ016349]!, KUN [barcode 608590]!, NAS [barcode NAS00353301]!, PE [barcode 00337996]!); Suichang, Guanshan (Fengping), 24 October 1965, *B.L. Qiu 1390* (paratypes: FUS, HHBG [barcode HZ016348]!). Fujian: Yanshan, on the slope, 800 m, 18 August 1958, *M.X. Nie & S.S. Lai 4290* (paratypes: FUS, IBSC [barcode 0319956]!, KUN [barcode 607929, 608566]!, LBG [barcode 00010452]!, PE [barcode 00394011]!, WUK [barcode WUK-265890]!).

**84. *Photiniazhejiangensis* P.L.Chiu, Acta Phytotax. Sin. 18(1): 97, pl. 2. 1980.**

≡ *Pourthiaeazhejiangensis* (P.L.Chiu) Iketani & H.Ohashi, J. Jap. Bot. 66(6): 354. 1991.

≡ *Pyruszhejiangensis* (P.L.Chiu) M.F.Fay & Christenh., Global Fl. 4: 126. 2018.

**Type**: China. Zhejiang: Jingning County, Gulou, 13 November 1958, *S.Y. Zhang 4505* (holotype: HHBG [specimen accession no. 10236]!, isotypes: KUN [barcode 0642805, 0642806]!, PE [barcode 00020624, 00020625]!). Jingning, Yingchuan, 9 May 1959, *S.Y. Zhang 4920* (paratypes: HHBG [barcode HZ016561]!, KUN [barcode 0642803]!, NAS [barcode NAS00354331, NAS00354332]!, PE [barcode 00394064]!). Longquan County, Guanpuyang, 650 m, 26 July 1958, *S.Y. Zhang 3236* (paratypes: HHBG [barcode HZ016558]!, NAS [barcode NAS00354333, NAS00354334]!, PE [barcode 00394365, 00394067, 00394068, 00394069)!]; ibidem, 9 November 1959, *S.Y. Zhang 7390* (paratypes: HHBG [barcode HZ016559]!, KUN [barcode 0642804]!, PE [barcode 00394006]!); ibidem, 2 December 1968, *Z.G. Mao 10720* (paratype: HHBG [barcode HZ016560]!). Taishun County, on the way from Liguang to Huangshikeng, 120 m, 27 November 1958, *Zhejiang Plant Resource Expedition 23822* (paratypes: HHBG [barcode HZ016556]!, NAS [barcode NAS00354335, NAS00354337, NAS00354338]!).

**85. *Photiniawuyishanensis* Z.X.Yu, J. Jiangxi Agric. Univ. 9(1): 5. 1982.**

**Type**: China. Jiangxi: Yanshan, Wuyishan, Xikeng, 1000 m, 26 April 1975, *Raowu Expedition Team 437* (holotype: JXAU; isotypes: LBG [barcode 00010454]!, LBG [barcode 00010455]!); Wuyishan, Dongkeng, 800 m, 11 May 1975, *Raowu Expedition 260* (paratypes: JXAU [barcode JXAU0003629]!, LBG [barcode 00010394]!, LBG [barcode 00010456, 00010457]!); ibidem, 810 m, 21 October 1981, *Q.G. Zhang W001* (paratypes: JXAU [barcode JXAU0003627, JXAU0003628]!); ibidem, 1,000 m, 24 October 1981, *Q.G. Zhang W030* (paratypes: JXAU [barcode JXAU0003626, JXAU0003630]!); Yushan: Longshou, Huaiyu Shan, 450 m. 19 September 1977, *Huaiyushan Forest Vegetation Expedition 220* (paratypes: JXAU, LBG [barcode 00010448, 00010449]!).

**86. *Photiniadabeishanensis* M.B.Deng & G.Yao, Bull. Nanjing Bot. Gard. Mem. Sun Yat Sen 1984–1985: 126. 1986.**

**Type**: China. Anhui: Jinzhai County, Baimazhai Forest Farm, 1,100 m, 17 May 1984, *M.B. Deng 81741* (lectotype, designated by Liu and Hong (2017: 19): NAS [barcode NAS00072797]!; isolectotype: NAS [barcode NAS00072798]!). Baimazhai Forest Farm, Zaoqian’ao, 1,500 m, 18 May 1984, *G. Yao 8988* (paratypes: NAS [barcode NAS00354378, NAS00354379]!).

**87. *Photiniamagnoliifolia* Z.H.Chen, J. Zhejiang Forest. Coll. 3: 35. 1986.**

**Type**: China. Zhejiang: Lin’an County, Qingshan Reservoir, 35 m, 15 April 1984, *Z.H. Chen & L.H. Ren 84006* (holotype: HHBG). Lin’an: Qingshan Reservoir, 24 July 1985, *Z.H. Chen & L.H. Ren 85003* (paratype: HHBG).

**88. *Photiniasubparvifolia* Y.K.Li & X.M.Wang, Bull. Bot. Res., Harbin 6(4): 108. 1986.**

≡ Photiniaparvifolia(E.Pritz. ex Diels)C.K.Schneid.var.subparvifolia (Y.K.Li & X.M.Wang) L.T.Lu & C.L.Li, Acta Phytotax. Sin. 38(3): 278. 2000.

**Type**: China. Guizhou: Libo, Jidong, 550 m, 17 April 1983, *X.M. Wang 191* (holotype: HGAS; isotype: PE [barcode 01432749]!).

**89. *Photiniazhijiangensis* T.C.Ku, Acta Phytotax. Sin. 31(2): 192. 1993.**

≡ *Pyruszhijiangensis* (T.C.Ku) M.F.Fay & Christenh., Global Fl. 4: 126. 2018.

**Type**: China. Hunan: Zhijiang County, Dashu’ao Xiang, Zhupo village, 600 m, 17 October 1988, *Wulingshan Expedition 2302* (holotype: PE [barcode 01896033]!; isotype: IBSC [barcode 0344337]!). nom. illeg. (referring to Liu and Hong (2017)).

**90. Photiniavillosa(Thunb.)DC.var.glabricalycina L.T.Lu & C.L.Li, Acta Phytotax. Sin. 38(3): 279. 2000.**

**Type**: China. Jiangxi: Shangyou, Wuzhifeng, 1 May 1972, *Jiangxi Expedition Team 1002* (holotype: PE [barcode 00337886]!; isotype: PE [barcode 01641097]!); Anfu, Wugong Shan, Kenzhichang, Yantian, the foot of the mountain, 10 August 1959, *S.S. Lai 1765* (paratypes: KUN [barcode 608499]!, LBG [barcode 00010381]!, PE [barcode 00337906]!); Ji’an, Donggu, Yingxu, Yangtian Shan, the forest edge, 16 May 1970, *s. coll. 272* (paratype: PE [barcode 00337923]!); Jinggangshan, along road between Xiaojing and Ciping, on the slope, 860 m, 13 October 1971, *Jiangxi Expedition 894* (paratypes: PE [barcode 00337912, 00337913]!); Nanfeng, Qia Cun, roadsides in the bushes, 23 April 1958, *M.X. Nie 2265* (paratypes: LBG [barcode 00010363]!, LBG [barcode 00014991]!, PE [barcode 00337934]!); ibidem, roadsides in the bushes, 25 April 1958, *M.X. Nie & S.S. Lai 2350* (paratypes: KUN [barcode 608524]!, LBG [barcode 00014974, 00014975]!, PE [barcode 00337933]!); Pingxiang, Xindian, sunny slope on the forest edge of *Camelliaoleifera* plantation, 300 m, 28 April 1954, *W.T. Wang 28* (paratype: PE [barcode 00337915]!); ibidem, sunny slope on the forest edge of *Camelliaoleifera* plantation, 300 m, 28 April 1954, *W.T. Wang 29* (paratype: PE [barcode 00337914]!); Ruijin: Liantang, longwangting, roadsides in the woods, 9 August 1958, *Q.M. Hu 4148* (paratypes: IBSC [barcode 0335603]!, KUN [barcode 608607]!, KUN [barcode 608539]!, LBG [barcode 00010431]!, PE [barcode 00337929]!); Xunwu, along road between Zhonghe and Guizhumao, 500 m, 1958, *Q.M. Hu 1252* (paratypes: IBSC [barcode 0335623]!, KUN [barcode 608525]!, LBG [barcode 00014988]!, PE [barcode 00337918]!); Xunwu, the valley in the east of Guizhumao, 700 m, 1 May 1958, *Q.M. Hu 1462* (paratypes: IBSC [barcode 0319949]!, KUN [barcode 608518]!, LBG [barcode 00010412]!, PE [barcode 00337931]!); Xunwu, vicinity of Guizhumao Forest Farm, 700 m, 3 May 1958, *Q.M. Hu 1584* (paratypes: KUN [barcode 608536, 608517], LBG [barcode 00010386]!, PE [barcode 00337930, 00337932]!). Guangxi: Lingui, Wantian, 120 m, 19 April 1955, *C.F. Liang 31703* (paratypes: IBK [barcode IBK00062287]!, IBSC [barcode 0319934]!, PE [barcode 00337965]!, SZ [barcode 00197053]!); Xing’an, Simen, May 1953, *Guangxi Team 4* (paratypes: PE [barcode 00337963, 00337964, 00337966, 01641095]!); Huajiang, 27 May 1953, *Guangxi Team 841* (paratype: PE [barcode 00337968]!). Guizhou: Leishan, vicinity of Wudong, 950 m, 6 May 1959, *South Guizhou Expedition 1310* (paratypes: KUN [barcode 608544]!, NAS [barcode NAS00354188, NAS00354321]!, PE [barcode 00337961, 01641101]!); Leishan, Xijiang, Leigongping, in the bushes on the slope, 1,100 m, 22 May 1959, *South Guizhou Expedition 2107* (paratypes: HGAS [barcode 021267]!, PE [barcode 00337869, 00337955]!); Leishan, Taoyao, Jieli, in the bushes on the slope, 108°8'20"E, 26°22'30"N, 950 m, 21 May 1965, *Z.B. Jian et al. 50082* (paratypes: HGAS [barcode 021282], KUN [barcode 608543], PE [barcode 00337958, 01498535]); Leishan, Wudong, Xiaoshuiyan, in the woods on the slope, 26°21'30"N, 108°9'E, 1,140 m, 22 May 1965, *Z.B. Jian et al. 50167* (paratypes: KUN [barcode 608541]!, PE [barcode 00337962, 01498532]!); Rongjiang, Bakai, Jihua, Baila, vicinity of Shangbaila, on the slope, 900 m, 25°39'30"N, 108°14'20"E, 8 August 1965, *Z.B. Jian et al. 51682* (paratypes: HGAS [barcode 021277]!, KUN [barcode 608655]!, PE [barcode 00337957, 01498533]!]); Tianzhu, 15 June 1937, *B.Q. Zhong 1090* (paratypes: PE [barcode 00337956, 00337959]!). Hunan: Hongjiang, Qianyang, District One or Two, 18 April 1953, *Anjiang Agricultural School 182* [paratypes: IBSC [barcode 0335715]!, PE [barcode 00337947]!]; ibidem, 12 August 1953, *Anjiang Agricultural School 645* (paratypes: IBSC [barcode 0319171]!, PE [barcode 00337943, 00337944]!). Jiangsu: Lianyungang, Yuntai Shan, Liuhe, 80 m, 27 August 1958, *F.X. Liu et al. 10848* (paratypes: IBSC [barcode 0335666, 0335668]!, NAS [barcode NAS00116611], PE [barcode 00337887, 00337888, 00337890, 00337891]!); Qiaya, Shendongcun, 260 m, 21 June 1956, *F.X. Liu et al. 2171* (paratypes: HHBG [barcode HZ016566]!, IBSC [barcode 0335662]!, NAS [barcode NAS00116596]!, PE [barcode 00337889]!). Zhejiang: Lin’an, Changhua, the tea plantation of Longtang Shan, in the stone crevices of the valley, 217 m, 2 October 1957, *X.Y. He 29819* (paratypes: HHBG [barcode HZ016611]!, NAS [barcode NAS00354049]!, PE [barcode 00337896]!); Ningbo, *s. coll. 1536* (paratype: PE); Yinxian, *G.R. Chen 2265* (paratypes: PE [barcode 00337893, 00337897]!).

**91. *Photiniasorbifolia* W.B.Liao & W.Guo, Ann. Bot. Fenn. 47(5): 394. 2010.**

≡ *Pourthiaeasorbifolia* (W.B.Liao & W.Guo) B.B.Liu & D.Y.Hong, Phytotaxa 325(1): 18. 2017.

**Type**: China. Hunan: Xinhuang County, Tianlei Shan Forest Farm, 760 m, 23 May 2008, *W. Guo & R.J. Shen 840* (holotype: SYS [barcode SYS00164356]!; isotypes: IBSC, SYS [barcode SYS00164354, SYS00164355]!). ibidem, 760 m, 10 May 2008, *W. Guo & R.J. Shen 841* (paratypes: IBSC, SYS); Mibei, Shiyangdong, Gualoupo, 900 m, 17 July 1988, *Wuling Expedition 909* (paratypes: IBSC [barcode 0335431]!, PE [barcode 01364106]!). Zhijiang: Longping, Baishuidong, 28 May 1959, *P.X. Tan 60949* (paratypes: IBK [barcode IBK00062671]!, IBSC [barcode 0317981]!)].

#### Naked names:

**1. *Photinialatouchei* Franch. ex Cardot, Bull. Mus. Natl. Hist. Nat. xxvi. 570, 1920. nom. nud.**

**2. *Photiniavariabilis* Hemsl., J. Linn. Soc., Bot. 23: 263. 1887.**

≡ *Pourthiaeavariabilis* (Hemsl.) Palib., Trudy Imp. S.-Peterburgsk. Bot. Sada xvii. I. 76. 1899. nom. nud.

**3. *Pourthiaealanata* Nakai, Bull. Natl. Sci. Mus., Tokyo No. 31, 62. 1952. nom. nud.**

**4. Pourthiaealaevis(Thunb.)Koidz.var.attenuata Nakai, Bull. Natl. Sci. Mus., Tokyo No. 31, 62. 1952. nom. nud.**

**5. *Rhaphiolepislaevis* Lodd. ex G.Don, Gen. Hist. 2: 602. 1832. nom. nud.**

#### Invalid names:

**Photiniavillosa(Thunb.)DC.var.typica C.K.Schneid., Ill. Handb. Laubholzk. i. 711. 1906.**

≡ Pourthiaeavillosa(Thunb.)Decne.var.typica (C.K.Schneid.) Nakai, Bot. Mag. (Tokyo) 30: 24. 1916. nom. inval.
